# Nuclear Factor κB Activation in a Type V Pityriasis Rubra Pilaris Patient Harboring Multiple *CARD14* Variants

**DOI:** 10.3389/fimmu.2018.01564

**Published:** 2018-07-03

**Authors:** Judit Danis, Anikó Göblös, Brigitta Gál, Adrienn Sulák, Katalin Farkas, Dóra Török, Erika Varga, Irma Korom, Lajos Kemény, Márta Széll, Zsuzsanna Bata-Csörgö, Nikoletta Nagy

**Affiliations:** ^1^Department of Dermatology and Allergology, University of Szeged, Szeged, Hungary; ^2^MTA-SZTE Dermatological Research Group, University of Szeged, Szeged, Hungary; ^3^Department of Medical Genetics, University of Szeged, Szeged, Hungary

**Keywords:** pityriasis rubra pilaris, psoriasis, nuclear factor κB signaling pathway, *CARD14* gene, inflammation

## Abstract

Pityriasis rubra pilaris (PRP) is a rare papulosquamous skin disorder, which is phenotypically related to psoriasis. Some familial PRP cases show autosomal dominant inheritance due to *CARD14* mutations leading to increased nuclear factor κB (NFκB) activation. Moreover, *CARD14* polymorphisms have also been implicated in sporadic PRP. A Hungarian PRP patient with childhood onset disease showing worsening of the symptoms in adulthood with poor therapeutic response underwent genetic screening for the CARD14 gene, revealing four genetic variants (rs117918077, rs2066964, rs28674001, and rs11652075). To confirm that the identified genetic variants would result in altered NFκB activity in the patient, functional studies were carried out. Immunofluorescent staining of the NFκB p65 subunit and NFκB-luciferase reporter assay demonstrated significantly increased NFκB activity in skin samples and keratinocytes from the PRP patient compared to healthy samples. Characterization of the cytokine profile of the keratinocytes and peripheral blood mononuclear cells demonstrated that the higher NFκB activation in PRP cells induces enhanced responses to inflammatory stimuli. These higher inflammatory reactions could not be explained solely by the observed *CARD14* or other inflammation-related gene variants (determined by whole exome sequencing). Thus our study indicates the importance of investigations on other genetic factors related to PRP and their further functional characterization to bring us closer to the understanding of cellular and molecular background of disease pathogenesis.

## Introduction

Pityriasis rubra pilaris (PRP; OMIM 173200) is a rare papulosquamous skin disorder, which is characterized by keratotic follicular papules, orange-red colored exfoliative dermatitis embracing "sparing islands" of normal skin and palmoplantar hyperkeratosis (1–4). PRP is usually self-limiting and the symptoms resolve within a few years after the onset of the disease (5). Histologically, PRP is characterized by alternating ortho- and parakeratosis oriented in vertical and horizontal directions ("checkerboard pattern"), irregular acanthosis, follicular plugging, perivascular lymphocytic infiltration of the dermis, and absence of neutrophils in the epidermis (1, 4, 6). Most cases of PRP are sporadic with multifactorial etiology; however, familiar forms were also described. Type V atypical juvenile variant accounts for approximately 5% of PRP cases with an early onset and prolonged course, with usually familial origin due to gain-of-function mutations in the *caspase recruitment domain family member 14* (*CARD14*) gene (3, 4, 6, 7). CARD14 protein is predominantly expressed in the skin, and plays an important role in regulation of skin inflammation by activating nuclear factor κB (NFκB) signaling. Certain *CARD14* mutations are thought to cause abnormal inflammatory response and thus, contribute to the development of PRP (3, 8–10).

Pityriasis rubra pilaris is often misdiagnosed as psoriasis (11), a more common papulosquamous inflammatory disorder affects approximately 2% of the European populations (12). The acute form of psoriasis includes guttate, pustular, and erythrodermic variants, while the chronic form includes plaque-type and flexural variants (13). Histologically, psoriasis is featured by parakeratosis, thickened projections of the prickle cell layer of keratinocytes, absence of the granular layer and polymorphonuclear leukocytic and lymphocytic infiltrates in the dermis and epidermis (14, 15). Psoriasis also exhibits multifactorial etiology (16–19). Variants identified to date that confer susceptibility explain less than 20% of psoriasis cases (20, 21). The remaining 80% of cases is presumed to be the consequence of yet unidentified susceptibility loci and environmental factors (8).

Recent studies demonstrated that *CARD14* variants and NFκB activation mediated by mutant *CARD14* is implicated in the development of both PRP and psoriasis and its rare variants have also been implicated in the development of generalized pustular psoriasis (8, 22–24). Similarly to its homologous protein CARD11, activated CARD14 interacts with the CBM-complex, namely the B-cell chronic lymphocytic leukemia/lymphoma 10 protein, which activates the mucosa-associated lymphoid tissue lymphoma translocation protein 1 a key member in the downstream activation of the NFκB pathway (6, 8, 25, 26). Germline mutations in the members of this signaling cascade are implicated in a broad spectrum of immune disorders, including severe immune deficiencies, lymphoproliferative disorders, and immune-mediated skin diseases (3, 22, 25–29).

Here, we report the detailed investigation of a Hungarian patient who was diagnosed with familial PRP based on clinical and histological findings and had family members suffering from psoriasis. Besides the analysis of the patient’s genetic background, we examined the NFκB activity of the patient’s samples by multiple methods.

## Case Report

### PRP Patient

**Figure 1:**
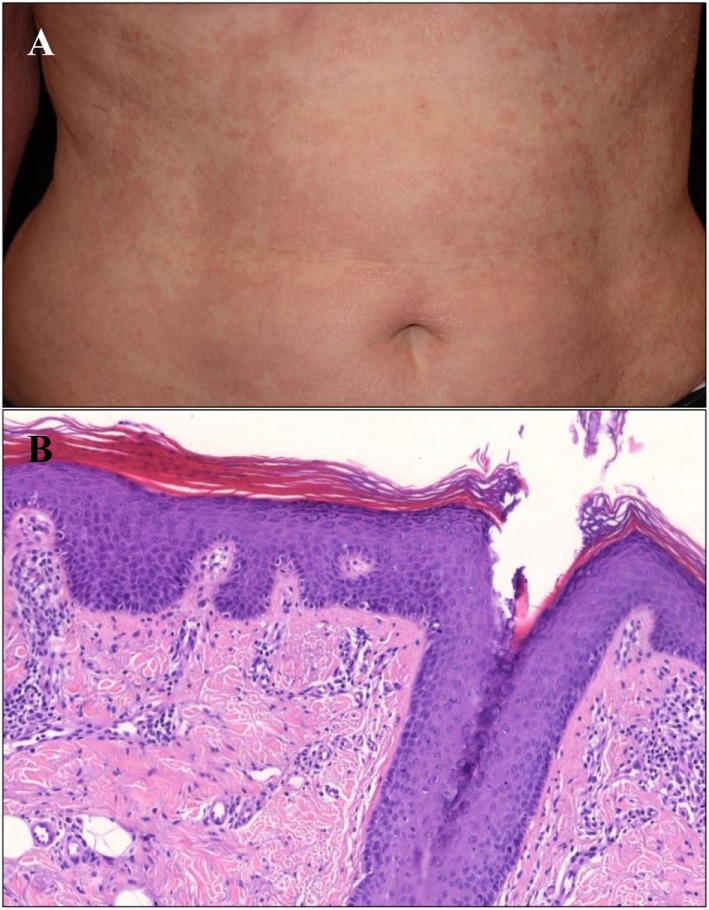
Clinical and histological image of the type V Pityriasis rubra pilaris (PRP) patient. **(A)** Erythematosus, hyperkeratotic confluent thin plaques with fine scales on the trunk of the patient. **(B)** There is follicular plugging with alternating hyper- and parakeratosis in the epidermis accompanied by granulosis, acanthosis, and mild spongiosis. A moderate lymphocytic infiltrate can be seen in the papillary dermis. The diagnosis of PRP has been confirmed by the histology (HE, digitally scanned slide).

The 61-year-old Hungarian woman was referred to the out-patient clinic of the Department of Dermatology and Allergology (Szeged; Hungary). Generalized erythroderma with mild infiltration and whitish fine scales were observed on her body (Figure 1A). The first onset of her skin symptoms occurred in childhood and she was under regular dermatological care for 28 years. Previous phototherapy and oral acitretin (25 mg/day) therapy were not effective, while methotrexate therapy was aborted due to serious side effects. Based on clinical and histological findings (Figure 1B) atypical PRP phenotype was diagnosed. Since the patient developed symptoms in early childhood turning into a chronic course with no sustained clearance, our patient was classified as a PRP type V patient. The patient was not aware of any family members affected by PRP; however, both her daughter and a grandchild had psoriasis and were also under regular dermatological care.

### Genetic Screening Identified *CARD14* Variants in the PRP Patient

The association between *CARD14* gene variants and PRP has recently been reported (3, 7). Direct sequencing of the *CARD14* coding regions of the PRP patient revealed three heterozygous missense variants: c.1641G/C p.Arg547Ser (rs2066964) in exon 14, c.2044C/T and p.Arg682Trp (rs117918077) in exon 17, and c.2458C/T p.Arg820Trp (rs11652075) in exon 20. The patient carried a splice site variant in homozygous form c.676-6G/A (rs28674001), located six nucleotides away from the 5′ end of exon 7. According to the results of analysis with pathogenicity prediction tools, the p.Arg682Trp missense variant is expected to be pathogenic, whereas the other three variants are expected to be benign.

### Increased NFκB Activity in the PRP Patient’s Skin Samples and Keratinocytes

**Figure 2:**
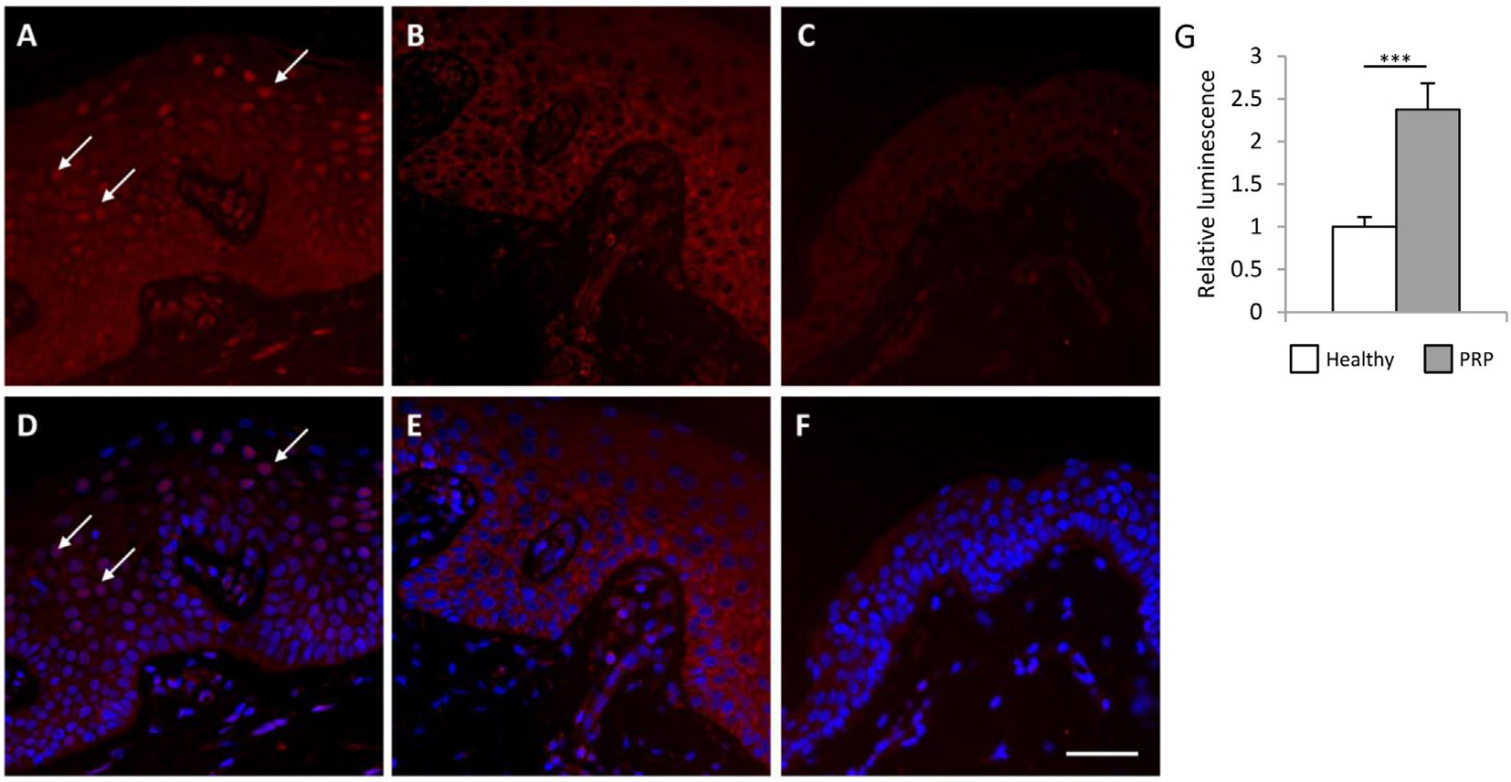
Lesional Pityriasis rubra pilaris (PRP) skin samples and keratinocytes exhibit higher nuclear factor κB (NFκB) activation compared to healthy samples. Skin-biopsy sections obtained from the PRP patient [lesional area: **(A,D)** non-lesional area **(B,E)**] and from a healthy individual **(C,F)** were stained with antibodies against the p65 subunit of NFκB. Nucleic staining of p65 comprises of active NFκB. Arrows indicate the p65-positive nuclei in the lesional PRP samples **(A–D)**, while no nuclear p65 staining was observed in the healthy and non-lesional PRP skin (Bar = 100 μm, healthy samples are representative for two individuals). Higher basal NFκB activity was observed in the cultured PRP keratinocytes compared to healthy keratinocytes by NFκB-luciferase reporter assay **(G)**. Cells were co-transfected with the pNFκB-luc Cis-Reporter Plasmid and the pGL4.75 control plasmid [hRluc/CMV]. The luciferase activity derived from the NFκB-luc plasmid was normalized to the activity of Renilla luciferase activity from the pGL4.75 [hRluc/CMV] plasmid, and compared to the luciferase activity of the healthy samples. Data are represented as mean ± SE, significance was determined by Student’s *t*-test; ***p < 0.001.

Previous studies suggested that *CARD14* variants contribute to the development of PRP by increasing the activity of the NFκB signaling pathway (3, 6). To investigate whether the detected *CARD14* variants in the investigated PRP patient increase NFκB activity, immunofluorescent (IF) staining was performed on paraffin-embedded samples of lesional and non-involved skin from the PRP patient and from healthy individuals (Figure 2). IF staining of the NFκB p65 subunit demonstrated that p65-positive nuclei (in 31.68% of epidermal cells) were present in the suprabasal epidermis of the lesional PRP skin, but were not present in the non-involved skin of the PRP patient or in the healthy skin samples. Moreover, analysis of fluorescence intensity revealed a stronger staining in PRP samples compared to healthy samples (Figure S2A in Supplementary Material). As an additional control group, patients with moderate-to-severe psoriasis vulgaris (PASI 22.4 and PASI 4.1) were also enrolled into the study, but p65-positive nuclei were not found either in lesional or non-lesional samples from psoriatic patients (Figures S4 and S5 in Supplementary Material). Among all examined sections, p65 appeared in the nuclei in the epidermis exclusively of the lesional skin from the PRP patient.

To further confirm increased NFκB activity in keratinocytes from the PRP patient, an NFκB-luciferase reporter assay was performed. The NFκB activity was significantly higher in the cultured keratinocytes of the PRP patient compared to healthy keratinocytes (Figure 2G).

In parallel, IF staining of the NFκB p65 subunit was also performed on peripheral blood mononuclear cells (PBMCs) derived from the PRP patient and healthy individuals (Figure S3 in Supplementary Material). Staining was more intense in the PBMCs of the PRP patient compared to cells of healthy individuals (Figure S2B in Supplementary Material). These results demonstrated increased NFκB activity in various cell types derived from the PRP patient.

### Both PRP PBMCs and Keratinocytes Express and Secrete Elevated Levels of Inflammatory Cytokines

To determine whether the increased NFκB activation had any functional consequence in the PRP patient, expression and secretion of NFκB-induced cytokines were determined.

**Figure 3:**
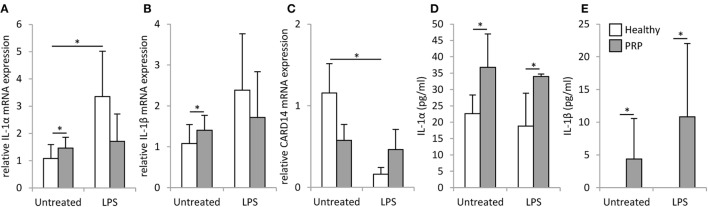
mRNA expression and secretion of inflammatory cytokines in keratinocytes. The mRNA expression of IL-1α **(A)** and IL-1β **(B)** and CARD14 **(C)** measured by RT-PCR and secretion of IL-1α **(D)** IL-1β **(E)** measured by ELISA were detected in keratinocytes derived from a healthy individual (white bars) and the Pityriasis rubra pilaris (gray bars) patient 6 h after treatment with 500 ng/ml LPS, time matched, and untreated samples served as controls. mRNA expression was normalized to the 18S rRNA expression and compared to the expression level of the untreated healthy control samples. Data are represented as mean ± SE of three experiments; *p < 0.05.

Keratinocytes of the PRP patient showed a tendency for higher basal mRNA expression and significantly higher secretion of interleukin (IL)-1α and IL-1β compared to healthy NHEKs (Figure 3). Upon LPS treatment in healthy keratinocytes mRNA expression induction was only detected for IL-1α (Figure 3A).

**Figure 4:**
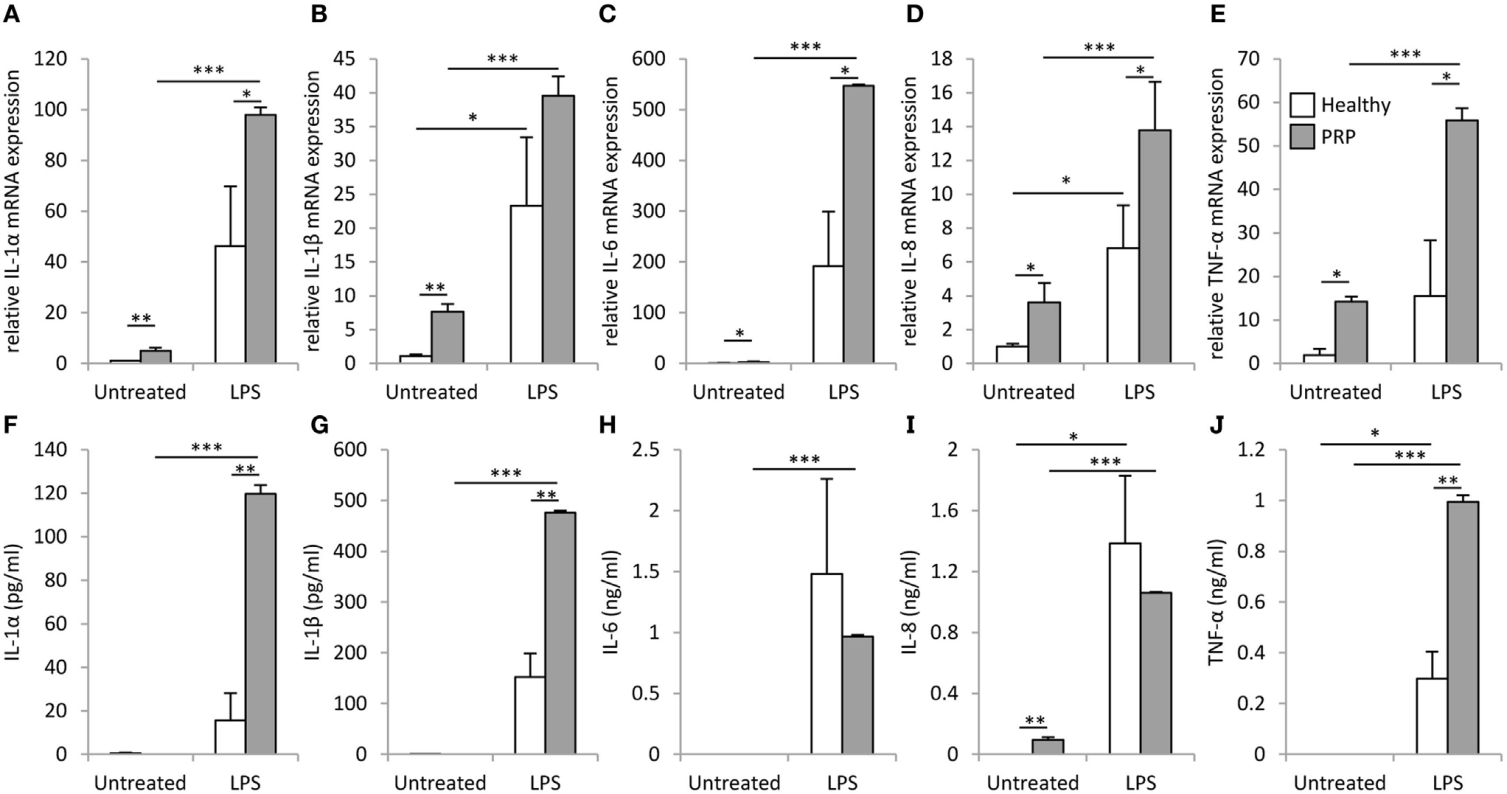
mRNA expression and secretion of inflammatory cytokines in peripheral blood mononuclear cells (PBMCs). IL-1α **(A)**, IL-1β **(B)**, IL-6 **(C)**, IL-8 **(D)**, and tumor necrosis factor (TNF)-α **(E)** mRNAs were detected in PBMCs derived from healthy (white) or Pityriasis rubra pilaris (PRP) (gray) cells 6 h after treatment with 100 ng/ml LPS. Secretion of IL-1α **(F)**, IL-1β **(G)**, IL-6 **(H)**, IL-8 **(I)**, and TNF-α **(J)** was detected for PBMCs derived from a healthy individuals (white, *n* = 3) or the PRP patient (gray, *n* = 1) 6 h after treatment with 100 ng/ml LPS. Data are represented as mean ± SE; statistical significance was determined by ANOVA *p < 0.05; **p < 0.01; and ***p < 0.001.

We found a significantly higher basal mRNA expression of IL-1α, IL-1β, IL-6, IL-8, and tumor necrosis factor (TNF)-α in PBMCs of the PRP patient compared to healthy control (Figure 4). In healthy PBMCs, LPS treatment significantly induced the mRNA expression of IL-1β and IL-8 compared to the untreated control samples, while in the PRP PBMCs significant induction (*p* < 0.001) was observed upon LPS treatment for all examined cytokines. The induction of IL-1α, IL-6, IL-8, and TNF-α was significantly (*p* < 0.05) higher in PRP cells compared to healthy control. The unstimulated PRP PBMCs secreted IL-8, while the secretion of the other cytokines was detected only after LPS treatment. IL-1α, IL-1β, and TNF-α were secreted at significantly higher levels in PRP cells compared to the PBMCs of healthy controls (Figure 4).

No significant difference was found between the basal CARD14 mRNA expression of healthy and PRP keratinocytes, but in healthy keratinocytes LPS treatment decreased CARD14 expression significantly, while CARD14 levels in PRP keratinocytes were not affected (Figure 3E).

## Materials and Methods

### Genetic Investigation

Blood samples were taken from investigated individuals (one PRP patient, psoriatic patients *n* = 2, and healthy individuals *n* = 5). Written informed consent was obtained from all investigated individuals to participate in the study and for the publication of this case report. The study was approved by the Human Investigation Review Board of the University of Szeged and complied with the ethical standards of research and in accordance with the Helsinki Declaration. Genomic DNA was isolated with QIAamp DNA Blood Mini Kit (QIAGEN, Hilden, Germany). After amplifying the coding regions and flanking introns of the *CARD14* gene using primer sequences displayed on the UCSC Genome Browser (30) and DNA sequencing was performed on amplification products.

The identified variants were analyzed with SIFT (31–36), PROVEAN (37–39), and PolyPhen-2 (40, 41) using online prediction tools for *in silico* analysis of their putative pathogenic role. Results of the genetic screening of all investigated individuals are described in Table S1 in Supplementary Material.

### Cell Culture and Sample Preparation

Peripheral blood mononuclear cells were isolated from whole blood of the PRP patient and three healthy individuals with a density-gradient centrifugation method using Ficoll-Paque. PBMCs were washed twice with phosphate-buffered saline (PBS) and seeded into 24-well plate at a density of 10^6^ cells/ml in RPMI 1650 media supplemented with 10% fetal bovine serum, 1% antibiotic/antimycotic solution (PAA Laboratories GmBH, Pasching, Austria), and 1% l-glutamine (PAA Laboratories). PBMCs were treated with 100 ng/ml LPS (Sigma Aldrich, St. Louis, MO, USA) for 6 and 24 h immediately after isolation. Time matched, untreated samples were used as controls, samples were harvested for RNA isolation and IF staining.

Human epidermal keratinocytes were isolated from two 6 mm punch biopsies derived from lesional skin of the lumbosacral region of the PRP patient and from a healthy skin specimen obtained from the Plastic Surgery Unit of our Department. The epidermis and the dermis were separated by overnight incubation in Dispase (Roche Diagnostics, Manheim, Germany), and keratinocytes were obtained after maceration in 0.25% trypsin. Cells were grown in 75 cm^2^ cell culture flasks for three passages in keratinocyte serum-free medium which contained epidermal growth factor and bovine pituitary extract (Gibco Keratinocyte SFM Kit; Life Technologies, Copenhagen, Denmark), supplemented with 1% antibiotic/antimycotic solution and 1% l-glutamine at 37°C in a humidified atmosphere with 5% CO_2_. Third passage keratinocytes were seeded into 12-well plate at a density of 150,000 cells/ml and 24 h later were treated with 500 ng/ml LPS for 6 h. Time matched, untreated samples were used as controls, samples were harvested for RNA isolation.

### NFκB-Luciferase Reporter Assay

Keratinocytes were seeded into 12-well plate at a density of 150,000 cells/ml in keratinocyte serum-free medium. 24 h later cells were transfected with the pNFκB-luc Cis-Reporter Plasmid (Stratagene) reporter construct vector and the pGL4.75 [hRluc/CMV] plasmid (Promega) internal control using the X-tremeGeneHP transfection reagent (Roche Diagnostics). 24 h after transfection, cells were washed in PBS and lysed in passive lysis buffer (Biotium, Hayward, CA, USA). Luciferase activity of the lysates was measured using the Firefly & Renilla Dual Luciferase Assay Kit (Biotium) and Thermo Luminoskan Ascent software (Thermo Scientific, Rockford, IL, USA), according to the manufacturer’s instructions. Luciferase activity derived from NFκB-luc plasmid was normalized to the activity of Renilla luciferase activity from the pGL4.75 [hRluc/CMV] plasmid. The transfection efficiency was similar in the PRP patient and healthy-control-derived keratinocytes, as determined by the transfection of a GFP reporter construct (Lonza, Basel, Switzerland) and flow cytometry.

### Immunofluorescent Staining

Peripheral blood mononuclear cells were harvested and 10^5^ cells were centrifuged onto a slide by a cytocentrifuge (Cytopro™, Wescor, Logan, UT, USA) and dried overnight at room temperature. The slides were fixed in 2% paraformaldehyde for 20 min. Skin specimens from healthy (*n* = 2), PRP (*n* = 1), and psoriatic patients (*n* = 2) were fixed in 4% buffered formaldehyde for 24 h. The tissue sample was subjected to paraffin embedding, and 4-μm-thick sections were placed on silanized slides, dewaxed in xylene for 3-times 5 min and rehydrated in decreasing concentrations of ethanol. Tissue retrieval was performed in citrate buffer (10 mM, pH 6.0).

Slides were exposed to 1% goat serum containing 1% bovine serum albumen (BSA) in PBS for 30 min. Slides were incubated overnight at 4°C with NFκB-p65 polyclonal antibody (rabbit IgG, 1:500; Santa Cruz Biotechnology, Dallas, TX, USA) in 1% BSA-PBS. Normal rabbit IgG antibody (Santa Cruz) was used as isotype control. Anti-rabbit Alexa Fluor 546 goat secondary antibody (Sigma Aldrich; 1:500) was applied for 2 h at room temperature. Cell nuclei were counterstained with 4,6-diamidino-2-phenylindole (DAPI, Sigma Aldrich, 1:500) and mounted with Fluoromount-G (Southern Biotech, Birmingham, AL, USA).

IF pictures were taken with the aid of a Zeiss Axio Imager fluorescent light microscope (Carl Zeiss MicroImaging) fitted with a Carl Zeiss AxioCam MRc5 camera. Fluorescence intensity of staining was quantified by the ImageJ software (developed by NIH).

### Real-Time RT-PCR

Peripheral blood mononuclear cells and keratinocytes were harvested using TRIzol^®^ Reagent (Invitrogen Corp., Carlsbad, CA, USA), following the manufacturer’s instructions. cDNA was synthesized from 1 µg total RNA using the iScript cDNA Synthesis Kit (Bio-Rad Laboratories, Hercules, CA, USA). Real-time RT-PCR experiments were carried out with the Universal Probe Library system (Roche Diagnostics) using a C1000 Touch Thermal Cycler (Bio-Rad Laboratories). Primers are listed as Table S2 in Supplementary Material.

### ELISA

Cell supernatants were centrifuged (8,000 rpm, 5 min, 4°C) to pellet cell debris and the amount of IL-1α, IL-1β, IL-6, IL-8, and TNF-α was determined by ELISA (R&D Systems, Minneapolis, MN, USA), according to the manufacturer’s instructions.

### Statistical Analysis

Experiments were carried out in triplicates; results are represented as mean ± SE. For statistical analysis, one-way ANOVA was used to compare more than two groups, and one-tailed, *T*-test was used to compare two groups. Statistical analysis was carried out with R software (Ver. 3.2.2.) with a significance level of *p* < 0.05.

## Discussion

CARD14 has a well-established immunomodulatory function: it plays an important role in the regulation of inflammation by activating the NFκB signaling pathway (3, 10) through its coiled-coil domain (42), and missense variants of this domain were shown to alter NFκB activation (8).

The *CARD14* variants identified in this study are located within different domains of the protein. The c.676-6 G/A splice variant is a frequent variant in the population (MAF = 0.34) located in the intronic region between the two exons encoding the NFκB activating coiled-coil domain. The p.Arg682Trp missense variant with a low minor allele frequency (MAF~0.01) is located on the SH3 domain. The more common p.Arg547Ser missense variant (MAF = 0.37) is located in the looping region between the coiled-coil domain and the PDZ domain, and the p.Arg820Trp (MAF = 0.35) missense variant is located on the catalytically inactive guanylate kinase domain.

Genetic variants of *CARD14* were described to affect NFκB activation in both psoriasis and PRP; however, the level of NFκB activation does not correlate with the severity of the disease or with the disease phenotype (8). *CARD14* mutations have been previously linked to type V PRP (7), which, due to the autoinflammatory pathogenic mechanism caused by mutation to *CARD14*, was recently characterized by Akiyama and colleagues as "autoinflammatory keratinization diseases" (43, 44). Association of the rs2066964 (encoding for p.Arg547Ser) mutation with psoriasis was found in some cohorts (8) and in patients with severe pustular psoriasis (45), and it was also detected with high frequency among sporadic PRP patients (6, 9). The rs117918077 (encoding for p.Arg682Trp) polymorphism of the *CARD14* gene has been associated with the development of psoriasis and it has also been previously identified in sporadic PRP patient (6, 8). The rs28674001 intron variant was described in PRP patients as neutral (6). The common missense variant rs11652075 polymorphism (encoding for p.Arg820Trp) showed association with psoriasis in several cohorts (8, 29), moreover, it was found in sporadic PRP patients (6, 9).

CARD14 is expressed predominantly in the skin (3). CARD14 expression was similar in both healthy and PRP keratinocytes, however, in contrast to healthy cells PRP keratinocytes showed no response to LPS, suggesting an aberrant regulation of CARD14 and subsequent processes in PRP. IF staining and NFκB-luciferase reporter assay demonstrated that NFκB activity was 2.5-fold higher in the PRP patient harboring all four *CARD14* variants than in healthy controls. Moreover, the higher NFκB activation in PRP cells was associated with higher cytokine responses both in PBMCs and cultured keratinocytes.

In previous studies the functional effects of the p.Arg682Trp and p.Arg547Ser variants have been investigated separately, using site-directed mutagenesis and a NFκB-luciferase reporter assay, which revealed no exerted effect of these variants on NFκB activation (6, 8). No previous functional data are available about the ability of the c.676-6G/A and p.Arg820Trp polymorphisms to influence NFκB activation.

Skin samples of PRP patients, not carrying any rare *CARD14* variants, also showed higher NFκB activation (46), which might be caused by the prolonged inflammatory milieu in the PRP patients body. The high population frequency of our observed polymorphisms and the previous finding on their inability to affect NFκB activation on their own (6, 8) suggest that their collective presence in the patient might not be the solely cause of the increased NFκB activation observed in patient samples. In line with these findings, our results showed increased NFκB positivity in skin samples and PMBCs of the PRP patient, which might be caused by the inflammatory phenotype of the patient. However, the patient’s keratinocytes, which were cultured for three passages in a non-inflamed environment, still maintained their higher NFκB activity. These results still suggest an inherited nature of the more intense NFκB responsiveness of our PRP patient.

Screening of the genes encoding members of the CBM signalosome complex and up- and downstream signaling by whole exome sequencing revealed no putative pathogenic sequences (Table S3 in Supplementary Material.), therefore, we exclude their involvement in the enhanced NFκB activity of this PRP patient. We assume that the cumulative effect of the *CARD14* genetic variants is one of the factors contributing to the increased NFκB activity of keratinocytes and PBMCs, which might lead to the development of PRP in the patient; however, the presence of yet unidentified variants in other genes cannot be excluded. Such variants could explain why only the investigated patient is affected by PRP while other family members are suffering from psoriasis. Unfortunately, family members of the patient have not agreed to undergo genetic screening, thus we were not able to identify these differentiating factors.

Pityriasis rubra pilaris is a complex inflammatory dermatosis with multifactorial etiology. Although CARD14 mutations identified in familial cases may provide an insight into the deregulated inflammatory processes in the disease, its exact pathogenesis remains to be elucidated. Our results highlight the possibility of other yet not identified inherited factors in the development of both PRP and psoriasis. High-throughput genetic analyses could shed light on other genes related to the disease.

## Ethics Statement

Written informed consent was obtained from all investigated individuals. The study was approved by the Human Investigation Review Board of the University of Szeged and complied with the ethical standards of research and in accordance with the Helsinki Declaration.

## Author Contributions

JD and AG designed and performed the experiments, analyzed the results, and drafted the manuscript. AS, KF, and DT performed the genetic analysis. BG and ZB-C diagnosed and enrolled the patient into the study. EV and IK contributed the histological samples and performed histological diagnosis. LK, ZB-C, and MS contributed to study design and critically reviewed the manuscript. NN contributed to study design and drafted the manuscript. All authors read and approved the final version of the manuscript.

## Conflict of Interest Statement

The authors declare that the research was conducted in the absence of any commercial or financial relationships that could be construed as a potential conflict of interest.
