# Supplementary material for: Nuclear Factor κB Activation in a Type V Pityriasis Rubra Pilaris Patient Harboring Multiple CARD14 Variants
Source: Front Immunol. 2018 Jul 3;9:1564. doi: 10.3389/fimmu.2018.01564 (PMC6037727; doi:10.3389/fimmu.2018.01564)
Supplement: Supplementary file 1 [file Data_Sheet_1.docx]

**Supplementary Tables**

**Supplementary Table 1. Genetic screening of *CARD14* variants in the PRP patient, psoriatic patients and healthy controls.**

| **CARD14 variant** | **PRP** | **PS1** | **PS2** | **Cont (biopsy)** | **Cont (PBMC)** | **Cont (PBMC)** | **Cont (PBMC)** | **Cont  (IF)** |
| --- | --- | --- | --- | --- | --- | --- | --- | --- |
| **c.676-6G/A rs28674001** | Homo- zygote | Wild type | Wild type | Wild type | Wild type | Wild type | Hetero-zygote | Wild type |
| **c.1641G/C p.Arg547Ser rs2066964** | Hetero- zygote | Wild type | Wild type | Wild type | Homo- zygote | Hetero- zygote | Hetero- zygote | Wild type |
| **c.2044C/T p.Arc682Trp rs117918077** | Hetero- zygote | Wild type | Wild type | Wild type | Wild type | Wild type | Wild type | Wild type |
| **c.2458C/T p.Arg820Trp rs11652075** | Hetero- zygote | Homo- zygote | Hetero- zygote | Hetero- zygote | Hetero- zygote | Homo- zygote | Hetero- zygote | Wild type |

**Supplementary Table 2. Primers used for real-time RT-PCR**

| Gene | | Primer sequence | UPL probe |
| --- | --- | --- | --- |
| 18S rRNS | FW | CGC TCC ACC AAC TAA GAA CG | 77 |
| 18S rRNS | REV | CTC AAC ACG GGA AAC CTC AC |  |
| TNF-α | FW | CAG CCT CTT CTC CTT CCT GAT | 29 |
| TNF-α | REV | GCC AGA GGG CTG ATT AGA GA |  |
| IL-1 α | FW | GGT TGA GTT TAA GCC AAT CCA | 6 |
| IL-1 α | REV | TGC TGA CCT AGG CTT GAT GA |  |
| IL-1β | FW | AAA GCT TGG TGA TGT CTG GTC | 10 |
| IL-1β | REV | AAA GGA CAT GGA GAA CAC CAC T |  |
| IL-6 | FW | CAG GAG CCC AGC TAT GAA CT | 45 |
| IL-6 | REV | GAA GGC AGC AGG CAA CAC |  |
| CARD14 | FW | gag ctc cta gac acg gca ga | 71 |
| CARD14 | REV | cga gac atc aag cct tcc ag |  |

**Supplementary Table 3. Whole exome sequencing does not reveal any putative pathogen variant in the genes of upstream and downstream NFκB pathways regulation in the PRP patient.**

Whole-exome sequencing service and bioinformatics analysis of the results was provided by the Xenovea Ltd. (Szeged, Hungary). The exomes were captured by Agilent SureSelect Human All Exon V6 kit (Agilent Technologies, Inc., Santa Clara, CA, USA) and the high-throughput sequencing was performed on Illumina NextSeq 500 system (Illumina Inc., San Diego, CA, USA).

The members of the CBM complex (*BCL10, CARD9, CARD10, CARD11, MALT1*) did not harbor any variants which result in altered protein products. All variants were detected in heterozygote genotype, and the detected variants were described as non-pathogenic or putative non-pathogenic. Other NFκB pathway members (*NFKBIA, NEMO, RELA, RELB, RIPK2, SYK*) were wild type, or harbored variants not leading to alterations of the protein product.

| **Gene** | **dbSNP ID** | **genotype** | **Consequence** |
| --- | --- | --- | --- |
| *BCL10* | rs2735594 | homozygote | upstream gene variant |
|  | rs11576939 | heterozygote | upstream gene variant |
|  | rs12037217 | heterozygote | upstream gene variant |
| *CARD10* | rs574761780 | heterozygote | inframe insertion |
| *CARD11* | rs1182136 | heterozygote | intron variant |
|  | rs1621509 | heterozygote | synonymous variant |
|  | rs6945582 | heterozygote | synonymous variant |
| *CARD9* | rs1135314 | heterozygote | 3'UTR variant |
|  | rs10781499 | heterozygote | downstream gene variant |
|  | rs4077515 | heterozygote | downstream gene variant |
| *NFKBIA* | rs8904 | heterozygote | 3'UTR variant |
|  | rs1957106 | heterozygote | synonymous variant |
| *TLR4* | rs2770148 | homozygote | intron variant |
| *MALT1* | wild type | | |
| *NEMO* | wild type | | |
| *RELA* | wild type | | |
| *RELB* | wild type | | |
| *RIPK2* | wild type | | |
| *SYK* | wild type | | |

**Supplementary Figures**

**
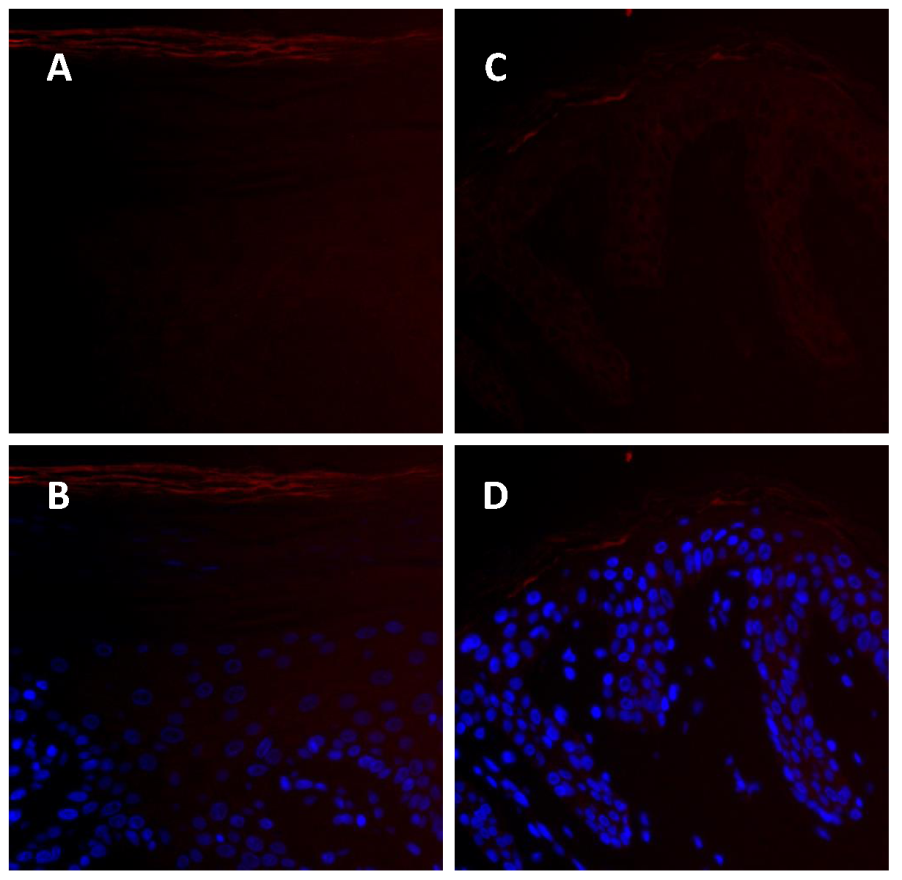
**

**Supplementary Figure 1. Isotype control staining in healthy and PRP skin.**

(a, b) PRP skin; (c, d) healthy skin. a, c without DAPI staining; b, d with DAPI staining. A minimal background staining can be detected in the stratum corneum and in the dermis. This level of staining, however, is much lower compared to the specific staining demonstrated in Figure 1.

**A B**

**
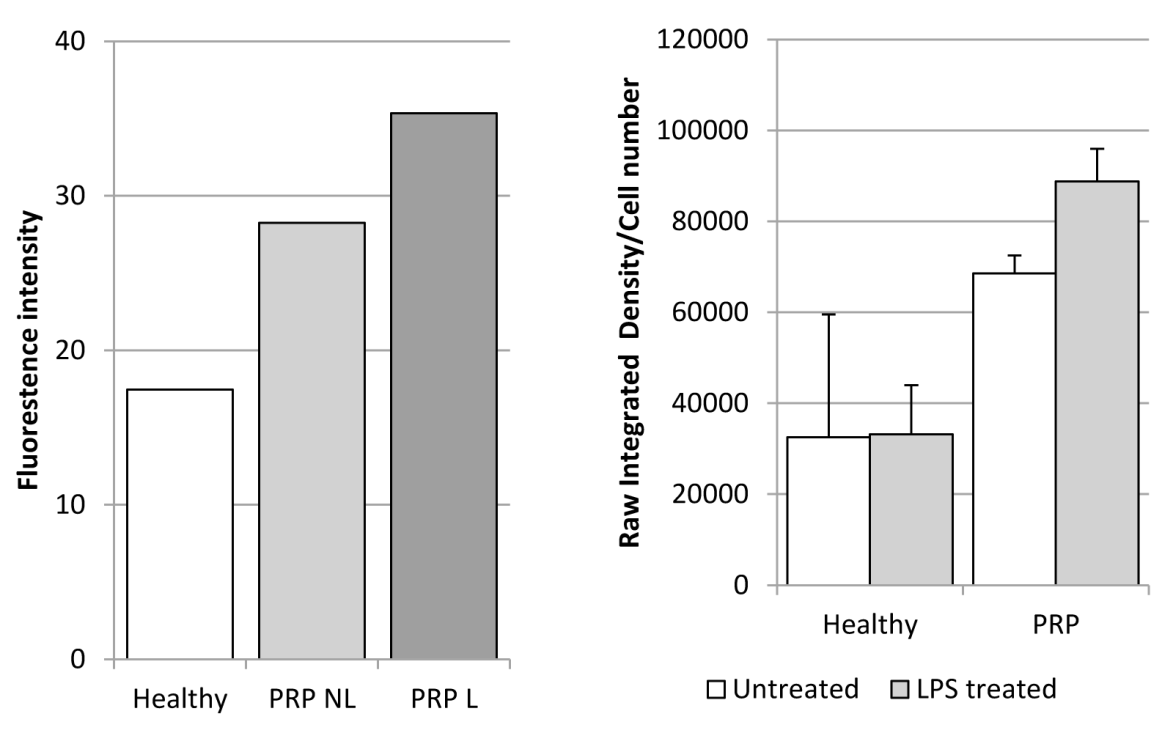
**

**Supplementary Figure 2. (A) Quantified fluorescent intensity of p65 staining in skin samples. (B) Fluorescence intensity in PBMCs upon LPS stimulation,** raw integrated density (determined by ImageJ for 5 field of view/individual) was normalized to cell number.


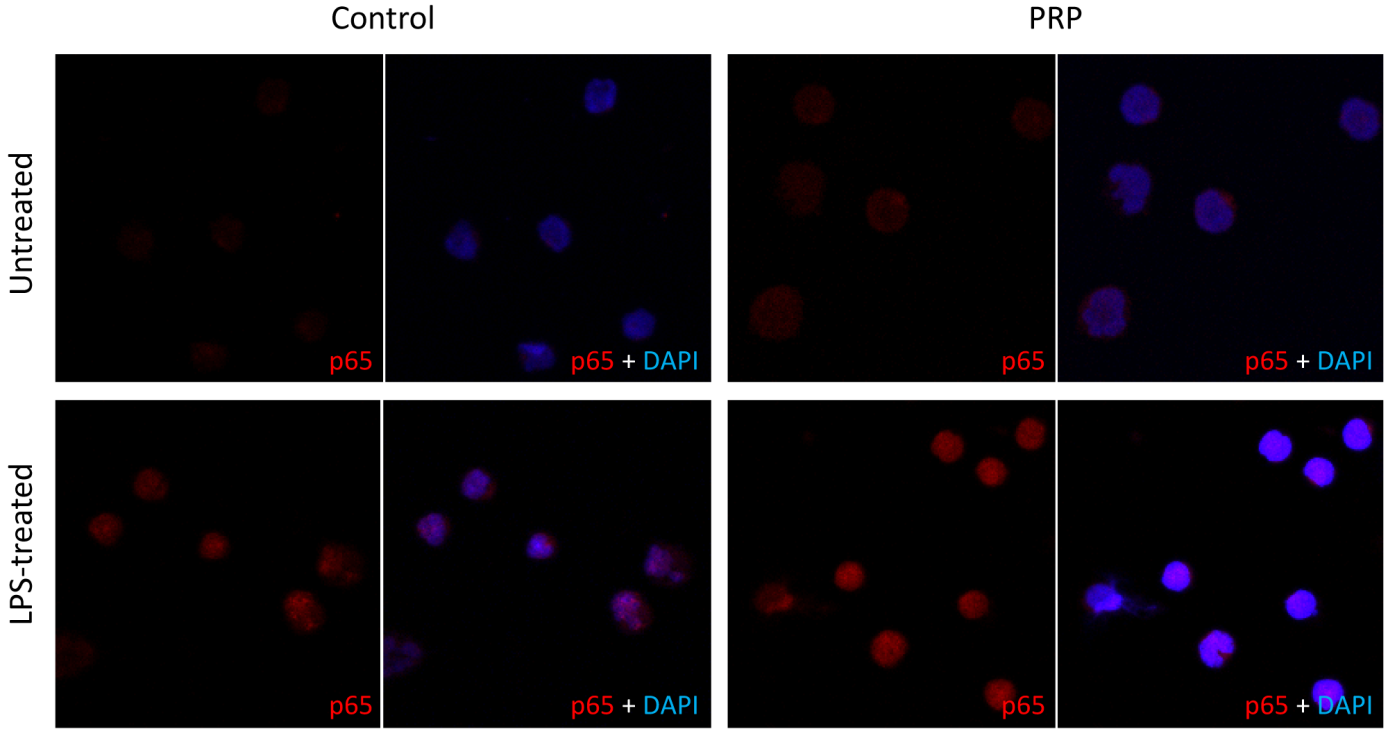


**Supplementary figure 3. Cellular distribution of the NFκB p65 subunit in LPS-induced PBMCs derived from the PRP patient and healthy individuals (figure is representative for n=3 healthy samples).** Magnification: 20X.

**
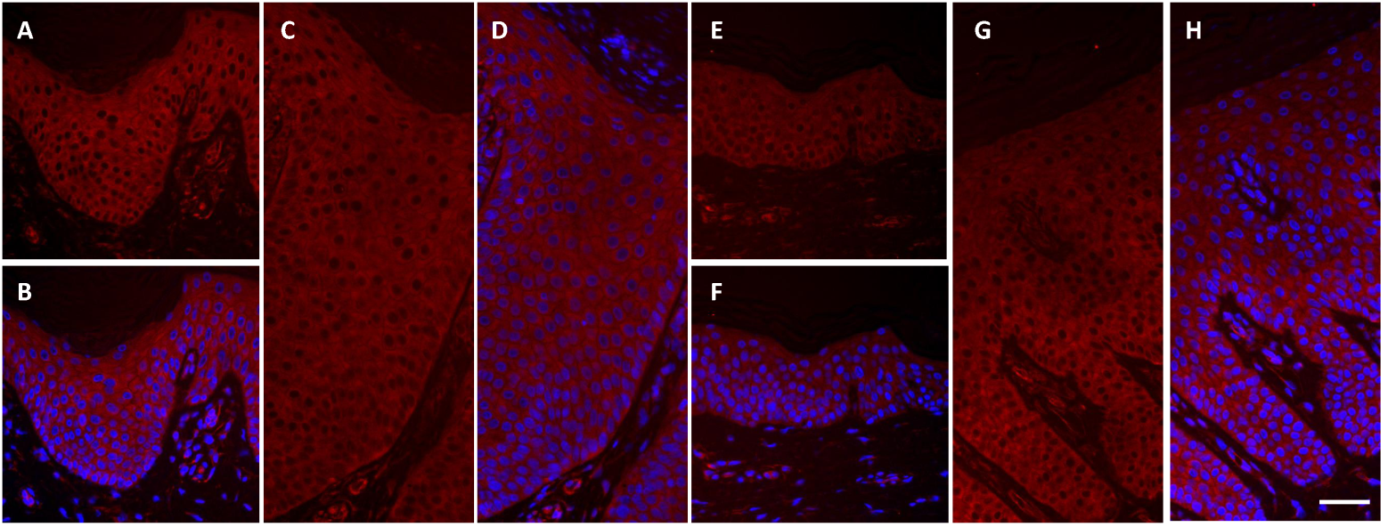
**

**Supplementary Figure 4. Immunofluorescent staining of the NFκB p65 subunit in psoriatic patients.** Skin-biopsy sections obtained from non-lesional skin (A, B) and lesional skin (C, D) of psoriatic patient I, and from non-lesional skin (E, F) and lesional skin (G, H) of psoriatic patient II were stained with antibodies against p65. Magnification: 20X. A, D, E and H were not stained with DAPI; B, C, F and G were stained with DAPI.

**
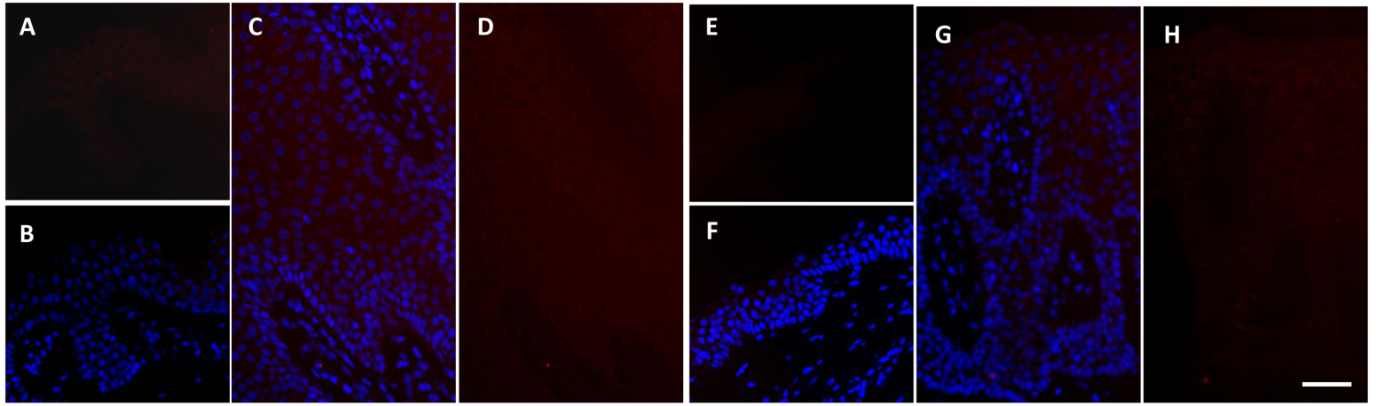
**

**Supplementary Figure 5. Isotype control staining in psoriatic skin.** (A, B) uninvolved skin of psoriatic patient I; (C, D) involved skin of psoriatic patient I; (E, F) uninvolved skin of psoriatic patient II; (G, H) uninvolved skin of psoriatic patient II. A, D, E and H show Alexa-Fluor-546; B, C, F and G were stained with DAPI. A minimal background staining can be detected in the dermis. This level of staining, however, is much lower than the specific staining demonstrated in Supplementary Figure 1.
